# From Then to now: Diversity, Equity, and Inclusion in the Association of Southeastern Biologists

**DOI:** 10.3389/fsoc.2021.755072

**Published:** 2021-10-27

**Authors:** Kimberly A. Hays, J. Christopher Havran, Matthew J. Heard, Ashley B. Morris, Loretta Ovueraye

**Affiliations:** ^1^ Department of Life Science, Dalton State College, Dalton, GA, United States; ^2^ Department of Biological Sciences, Campbell University, Buies Creek, NC, United States; ^3^ Department of Biology, Belmont University, Nashville, TN, United States; ^4^ Department of Biology, Furman University, Greenville, SC, United States; ^5^ Division of Workforce Programs and Professional Learning, Miami Dade College, Miami, FL, United States

**Keywords:** scientific society, diversity, equity, inclusion, racism, segregation

## Abstract

The Association of Southeastern Biologists was founded in 1937 with the goal of increasing the contact and collaboration between scientists in the southeastern United States (US). With the exception of two years during World War II and one year during the COVID-19 pandemic, the Association has met annually to promote research and education in the biological sciences by providing a student-friendly networking environment. In recent years, the Association has placed an increased focus on diversity, equity, and inclusion among elected and appointed leaders, among participants in the annual meeting, and in the development of funding and other opportunities for students. This work prompted us to review the history of our Association, including periods of racial segregation and inequity, and focus on our current efforts to promote access and inclusion by students and scientists from myriad underrepresented groups. In so doing, the past provides us with the opportunity to cast a vision for the future of the Association. In this paper, we seek to share the journey of the Association of Southeastern Biologists in this regard so that we may be transparent, exposing the missteps and amplifying the successes of our organization. We envision this work as a first step toward creating a more open and inclusive scientific community for the future.

## Introduction

Professional societies play a critical role in disseminating scientific discoveries and forging collaboration among scientists. These organizations typically fall into two categories: 1) national or international organizations with large meetings that move across a broad geographical area or 2) smaller statewide and regional meetings and organizations. To our knowledge, since its founding in 1937, the Association of Southeastern Biologists (ASB) has been the only scientific association that spans the entire Southeastern US and is one of the largest regional, multi-disciplinary biological associations in the country. The ASB operates with the goal of increasing contact and collaboration between scientists in the region, representing biologists from many sub-disciplines, including student, faculty, and non-academic professionals. While the ASB prides itself on being a working association with an active and engaged membership, the past several years have served as a period of reflection on our growth and values, specifically as they relate to diversity, equity, and inclusion. Upon reflection, we feel that the ASB, as a broad-reaching organization, is uniquely positioned to drive change not only within our own association but across academic institutions represented within the ASB, and our practice may serve as a model to other scientific societies.

**FIGURE 1:**
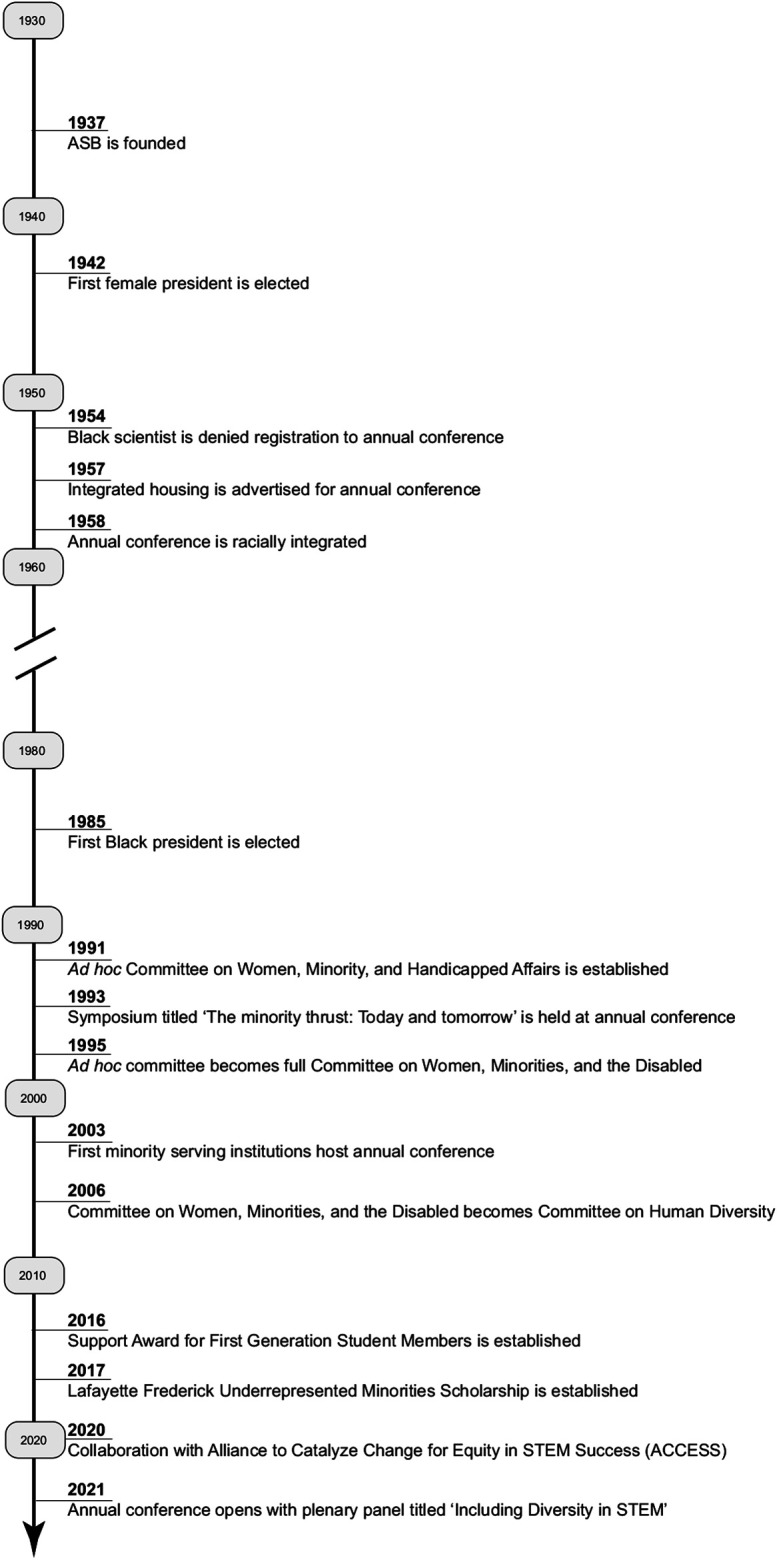
A reconstructed history of the Association of Southeastern Biologists (ASB) highlighting events covered in this perspective.

As we in the ASB leadership reflect on our history (Figure 1), we encounter members who championed change and diversity to drive the Association forward during periods of conflict and challenge. We also recognize that increased diversity in membership does not by definition mean equity and inclusion, which are key goals of our association. In our reflection and in this paper, we seek to share the journey of the ASB by examining: 1) our early history as an organization in the segregated South and the role of members in keeping us afloat while driving change, 2) our contemporary history in which we are actively working to build diversity, equity, and inclusion into all aspects of our association, and 3) our recommendations for current and future leadership of the ASB to ensure that our association is an open forum for all biologists in the Southeastern US. For the purposes of this narrative, the terms minorities or underrepresented groups refer to those individuals who were historically excluded from participating in the scientific community as a consequence of their gender, race, ethnicity, sexual identity or ability; however, the historical records of the ASB often do not explicitly define these terms.

## Early History

To better foster communication between biologists in the Southeastern US, the ASB was officially established as a scientific association at an invitational meeting at the University of Georgia in 1937 (Boyd, 1957). The inaugural meeting consisted of oral presentations on all aspects of biology, primarily from scientists in states that shared borders with Georgia (Boyd, 1957). The Association continued to meet annually in the spring, hosted by institutions in cities across the Southeastern US. The program evolved to include banquets, field trips, and awards to recognize outstanding research and meritorious teaching. None of the early meetings of the ASB were hosted by minority-serving institutions and no demographic records of meeting participants were kept in the early years of meeting.

Histories of the ASB (Boyd, 1957; Young, 1963; Flory, 1987) acknowledge the contribution of women to various aspects of association governance, with one taking on an outstanding role during the early years of the ASB. From 1943 to 1945, annual meetings of the ASB were put on hold during World War II (Young, 1963). At the last annual meeting before the war, Dr. Mary Stuart MacDougall of Agnes Scott College was elected President of the ASB. Dr. MacDougall was the sixth president of the ASB and the first woman to hold this position. With the assistance of the elected Secretary-Treasurer, Dr. Martin D. Young, Dr. MacDougall maintained the ASB while meetings were on hiatus (Boyd, 1957). She accomplished this through the reduction of annual dues and sending out newsletters to the membership, updating them on ASB affairs and the activities of members in uniform (Young, 1963). Dr. MacDougall’s efforts to maintain the Association at a time when it very well could have dissolved have been applauded in several histories of the ASB (Boyd 1957; Young 1963). In his summary of the 25-years history of the ASB, Young (1963) expounded on the historical role of women in the association by mentioning how “women have served on various important committees and in general have had a prominent voice in the operation of the society”. He mentions that this is “a shining example of integration”.

In the 1950s, adherence to Jim Crow Laws would, unfortunately, limit participation of Black scientists in ASB functions. In 1954, Dr. James H.M. Henderson, a plant physiologist from Tuskegee University, was denied registration to the annual meeting held in Baton Rouge, Louisiana (Frederick, 2012). Dr. Henderson was Black, and the onsite representatives would not allow him to attend the banquet (Frederick, 2012). Dr. Henderson never participated in a subsequent ASB meeting, but continued his illustrious and internationally-recognized career (Henderson, 2001). Beginning in 1954, the annual meeting banquet would temporarily be put on hold based on the rationale that it was difficult to schedule the banquet around other conference functions (Boyd, 1957). While there is some lack of clarity related to the chronology of events in the 1950s, association records and oral accounts clearly indicate that, during this decade, the ASB followed Jim Crow laws that prevented communal eating. These choices prevented Black members from attending the banquet, which is now a pivotal component of the annual meeting. Beginning in 1957, notices began to appear in the ASB Bulletin about the location of integrated housing facilities for annual meetings (About our Tallahassee Meeting, 1957).

Racial integration of the ASB would not occur until the late 1950s. In 1958, Dr. Lafayette Frederick, a Black plant biologist then at Southern University in Baton Rouge, Louisiana, was recommended to attend the annual meeting of the ASB in Gainesville, Florida (Frederick, 2012). He took three students and two faculty members to the conference and secured housing in private homes for his female students (Frederick, 2012). He and his colleagues presented their research and attended the awards ceremony in an auditorium (Frederick, 2012). This event is recognized as the point at which the Association was racially integrated. Dr. Frederick continued to bring his students and colleagues to ASB meetings. Despite resistance that they faced during at least one social function, Dr. Frederick insisted that his students and colleagues continue to attend meetings of the ASB (Frederick and Williams, 2017). He persisted with his efforts and was eventually elected as ASB’s first and only Black president in 1985 (Herr, 2012). Dr. Frederick would continue to be integral to the ASB’s efforts to promote diversity in the organization, attending annual meetings until his death in 2018.

## Contemporary History

A key turning point in the ASB’s efforts in diversity, equity, and inclusion came *via* the 1990 American Institute of Biological Sciences (AIBS) Conclave of Presidents. The event, held in Washington D.C., was attended by ASB President Frank Day. In his “A View from Here” column, Day (1990) stated “A specific societal issue that was extensively discussed at the conclave was the need to increase the involvement of minorities, women, and the disabled in our profession. Actions that ASB might consider are creation of a committee to address involvement of underrepresented groups and targeting local school kids with special programs in association with our annual meeting”. While there is no indication that engagement with schools occurred, this call for a committee to address the needs of underrepresented individuals spurred the Association into several years of concerted action.

ASB members were involved in organizing and presenting at the 41st Annual Meeting of AIBS in Richmond, Virginia. Included in this meeting were a symposium, roundtable, and reception focused on minorities in biology (AIBS, 1990). In that same year, AIBS conducted a survey of 30 AIBS member and cooperating societies, 14 other life science societies, and seven organizations from other disciplines. The survey focused on the number of members who were women or minorities, the ability of societies to identify these members, and inclusion of events for women and minorities at annual meetings or in other settings. ASB was included in this survey, reporting a total membership of approximately 1,200 members. ASB was one of 65% of societies that did not identify the number of female members and 97% of societies that did not identify the number of minority members. While this survey did not indicate if ASB hosted specific activities or committees for women or minorities, no historical records of those appear to exist until 1991 (Blockstein, 1990).

In 1991, ASB President Frank Day appointed an *ad hoc* committee on Women, Minority, and Handicapped Affairs that was tasked with organizing “activities and programs which encourage and enhance the involvement of underrepresented groups in biology” (Day, 1991). The original committee was composed of Dr. LaFayette Frederick (Chair; Howard University), Dr. Margaret Gilbert (Florida Southern College), Dr. Elizabeth Blood (University of South Carolina), Dr. Beverly Collins (Memphis State University), and Dr. Geraldine Twitty (Howard University). In 1993, the committee hosted its first organized event at the 54th Annual Meeting in Virginia Beach, Virginia. The symposium, “The Minority Thrust: Today and Tomorrow”, featured Dr. Clarice Gaylord, Deputy Director of the Environmental Equity Office with the U.S. Environmental Protection Agency. The committee scheduled symposia and workshops surrounding underrepresented groups in science, technology, engineering, and mathematics (STEM) fields fairly consistently through the late 2000s, at which time a shift was made to luncheons that often featured topical speakers.

Later in 1993, the ASB Place of Meeting Committee was tasked by then President Ken Marion to further the work of the Committee on Women, Minorities, and Handicapped Affairs by organizing an annual meeting at a minority-serving institution (Marion, 1993). Association records indicate that the committee transitioned from an *ad hoc* committee to a full committee in the mid-1990s, with the full committee being renamed as the Committee on Women, Minorities, and the Disabled (Committees, 1995).

In her message from the President, Dr. Pat Parr recounted the ASB’s participation in the 1999 National Science Foundation President’s Summit. According to Parr, the ASB and Ecological Society of America were the only two organizations of the 53 represented that had a committee focused on diversity and minority involvement. She stated, “Not only does ASB have wide representation from colleges, universities, non-academia, and disciplines, ASB has successfully demonstrated a commitment to support our students, improve education, recognize excellence, and encourage diversity” (Parr, 2000). When asked recently to reflect on the accomplishments of the committee in its first decade, Parr stated “What the committee achieved?—some positive but not enough, and not long lasting. We tried very hard to identify HBCU and community colleges in the area where ASB was meeting and send postcards and tried other ways to make personal contacts to get professors and students to attend” (P. Parr pers. comm., 2021).

In 2003, the ASB annual meeting was co-hosted by Howard University and Bowie State University in Washington, D.C., marking the first time that a minority-serving institution had done so. At that same meeting, the ASB Committee on Women, Minorities, and the Disabled hosted its first workshop focused on disability access titled “Accessibility of Online Teaching Materials”. To our knowledge, no further workshops or symposia have focused on disability access.

In 2006, the Committee on Women, Minorities, and the Disabled was renamed the Human Diversity Committee to better encompass the breadth of diversity represented by the ASB membership. One major focus of the Human Diversity committee was to increase participation of first-generation and underrepresented students in the annual meeting. In 2016, the committee began awarding the Support Awards for First-Generation Student Members of ASB, which provides support for winners to attend the annual meeting. In 2017, the committee worked with the ASB Executive Committee, Dr. Lafayette Frederick, Dr. Luther Williams, and the Frederick family to create the Lafayette Frederick Underrepresented Minorities Scholarship, which provides one student each year with meeting registration, attendance costs, membership, and travel expenses. Since the inception of these awards, the first-generation and Lafayette Frederick Underrepresented Minorities Scholarships have supported thirty and three students, respectively. Upon analysis, the ASB leadership realized that while these awards that help underrepresented students attend the annual meeting are a good starting point on our journey to creating a more diverse scientific society, more work can be done to advertise these awards to both students and mentors.

Building and maintaining a scientific society that is truly diverse, equitable, and inclusive requires continual work and strategic planning to be successful (Olzmann, 2020; Tulloch, 2020). Therefore, over the past few years we have begun directed efforts to prioritize creating a more open and inclusive environment at our annual meetings. In 2020, to help facilitate this change and to evaluate our current practices, the ASB Executive Committee entered into a collaboration with the Alliance to Catalyze Change for Equity in STEM Success (ACCESS) via co-principal investigator and member of the ASB Executive Committee, Dr. Veronica Segarra (High Point University). The goal of this effort was to determine how the ASB and other organizations select conference speakers that are representative of all members. Through this collaboration, ACCESS provided the ASB and other organizations with a list of best practices for how to foster inclusivity through plenary sessions at the annual meetings (Segarra et al., 2020).

Building on this work with ACCESS and their recommended best practices, the ASB Executive Committee chose to focus the plenary session of the 2021 annual meeting on diversity in STEM and how it relates to the ASB and biologists in the Southeastern US. The plenary session was titled “Including Diversity in STEM” (Association of Southeastern Biologists, 2021) and was moderated by an African American ASB Executive Committee member, Dr. Loretta Ovueraye, Vice Provost for Workforce Programs and Professional Learning at Miami Dade College. In the session, Dr. Ovueraye led a conversation with a group of ethnically and socially diverse individuals with science education and research backgrounds: Dr. Edward Moreira Bahnson from the University of North Carolina at Chapel Hill, Dr. Kelly Mack from the Association of American Colleges and Universities, Dr. Veronica Segarra from High Point University, and Dr. Selwyn Williams from Miami Dade College. Panelists discussed the meaning of diversity to them. Together, they described the particular disparities they see pertaining to diversity within biological institutions that limit our ability to recognize the range of diversity that already exists. This led to an exploration of the structures of higher education that have contributed to these systemic inequalities and the long-term consequences of ignoring diversity and inclusion in the STEM workforce. In a post-meeting survey, 45% of respondents indicated they were satisfied or very satisfied with the plenary session and no respondents indicated they were dissatisfied or very dissatisfied. One attendee commented, “ASB could make this available and become a leader in the efforts—there was so much realism and advice and perspective packed into this session. Truly, thank you for making this the opening session!”.

This conversation gave us a blueprint for a more inclusive environment within the ASB to address the larger societal issue of underrepresentation of minority groups in STEM. Attendees who have impactful experiences like this can then influence policies and practices surrounding diversity, equity, and inclusion at their own institutions.

## Recommendations Moving Forward

This manuscript provides the ASB leadership a starting point for casting a vision for the future of our association. The process of developing this document has allowed us to dig deeply into our history and take stock of our strengths and weaknesses. Based on the events and experiences represented here, we have several recommendations for the ASB leadership moving forward, including: 1) complete a comprehensive strategic plan for the Association that clearly integrates diversity, equity, and inclusion throughout; 2) pursue an external assessment of the Association’s diversity, equity, and inclusion activities; 3) increase engagement of members with disabilities to improve accessibility to both content and activities; 4) collaborate with HBCUs and other Minority Serving Institutions (MSIs) currently active in the ASB to strengthen and build additional partnerships; 5) engage first-generation and underrepresented students and their mentors in the application process for diversity, equity, and inclusion support awards; and 6) pursue external funding for mentorship activities.

## Conclusion

The ASB was established in the segregated South, and it is clear that past actions of the Association caused harm to established and incipient scientists. Lasting social change must come from within, and it is guided by the actions of individuals. As we move forward, we recognize that there are still many more steps that the ASB must take in order to be truly supportive of all of its members. These efforts include working to be actively anti-racist, to provide support and mentoring opportunities for historically excluded students and faculty, to value and champion diversity within our scientific society, and to fight for equality and inclusivity in our communities. To truly achieve these steps, we acknowledge that we need to continue to be reflective, build on the expertise of scientists and individuals working to address diversity, equity, and inclusion issues within our association and more broadly, and to honor the legacy of our past heroes and champions within the ASB, including Dr. Mary Stuart MacDougall, Dr. Lafayette Frederick, and others who helped break down barriers that prevented the ASB from being a truly inclusive organization. In closing, we invite all members of the ASB and ASB’s friends, partners, and affiliates throughout the Southeastern US to join us in these efforts.

## Data Availability

The original contributions presented in the study are included in the article/supplementary material, further inquiries can be directed to the corresponding author.
